# Molecular Cloning and Sexually Dimorphic Expression Analysis of *nanos2* in the Sea Urchin, *Mesocentrotus nudus*

**DOI:** 10.3390/ijms20112705

**Published:** 2019-06-01

**Authors:** Jian Zhang, Xiao Han, Jin Wang, Bing-Zheng Liu, Jin-Liang Wei, Wei-Jie Zhang, Zhi-Hui Sun, Ya-Qing Chang

**Affiliations:** Key Laboratory of Mariculture & Stock Enhancement in North China Sea, Ministry of Agriculture and Rural Affairs, Dalian Ocean University, Dalian 116023, China; zj7782133@163.com (J.Z.); im_hanxiao@163.com (X.H.); wangjin19980318@163.com (J.W.); lbz051014@163.com (B.-Z.L.); m15524575713@163.com (J.-L.W.); zhangweijie@dlou.edu.cn (W.-J.Z.)

**Keywords:** sea urchins, *nanos2*, germ cell, RNAi

## Abstract

Sea urchin (*Mesocentrotus nudus*) is an economically important mariculture species in China and the gonads are the solely edible parts to human. The molecular mechanisms of gonad development have attracted increasing attention in recent years. Although the *nanos2* gene has been identified as a germ cell marker in several invertebrates, little is known about *nanos2* in adult sea urchins. Hereinto, we report the characterization of *Mnnano2*, an *M. nudus nanos2* homology gene. *Mnnanos2* is a maternal factor and can be detected continuously during embryogenesis and early ontogeny. Real-time quantitative PCR (RT-qPCR) and section in situ hybridization (ISH) analysis revealed a dynamic and sexually dimorphic expression pattern of *Mnnano2* in the gonads. Its expression reached the maximal level at Stage 2 along with the gonad development in both ovary and testis. In the ovary, *Mnnanos2* is specifically expressed in germ cells. In contrast, *Mnnanos2* is expressed in both nutritive phagocytes (NP) cells and male germ cells in testis. Moreover, knocking down of *Mnnanos2* by means of RNA interference (RNAi) reduced *nanos2* and *boule* expression but conversely increased the expression of *foxl2*. Therefore, our data suggest that *Mnnanos2* may serve as a female germ cell marker during gametogenesis and provide chances to uncover its function in adult sea urchin.

## 1. Introduction

The *Nanos* gene encodes an RNA-binding protein and was first identified as a determinant of the anterior-posterior axis in *Drosophila melanogaster* [1]. Several *nanos* homologous genes, including *Nanos1a*, *Nanos1b*, *Nanos2*, and *Nanos3*, have been characterized in diverse species since then [2,3,4,5,6]. Accordingly, their evolutionarily conserved roles in germ cell development have been revealed in both invertebrates [7] and vertebrates [8].

As a member of the *Nanos* gene family, *nanos2* has been identified as a germline stem cells (GSCs) marker that plays an important role in germ cell regeneration [6,9]. However, *nanos2* shows various expression patterns, functions, and regulatory mechanisms among different species. For instance, *Nanos2* is predominantly detected in male germ cells in Mammalia (e.g., mouse), and knockout of *Nanos2* leads to a complete loss of spermatogonia [8,10,11]. In Aves (e.g., chicken), *nanos2* is mainly expressed in testes and promotes the transformation of embryonic stem cells into male germ cells [12]. In contrast, the expression pattern of *nanos2* in fish shows non-uniformly species-specific characteristics. For example, *nanos2* is expressed in GSCs in zebrafish, but mainly restricted to undifferentiated spermatogonia in rainbow trout [9,13]. In Echinodermata, studies of the *nanos* gene have mainly focused on embryogenesis. For example, *nanos* is detected primarily in the posterior enterocoel during embryogenesis with a subsequently continuous expression in the germline in sea star (*Patiria miniate*) and is associated with decreases in the cell cycle of the primordial germ cell (PGC) [14]. In sea urchin (*Strongylocentrotus purpuratus*), *nanos2* is first detected in small micromeres at the 60-cell stage, and it is required for maintaining the small micromere descendants into the larval coelomic pouches [15,16]. However, the expression pattern in adult gonads of sea urchin is still unclear.

*Mesocentrotus nudus* is an echinoid of the Strongylocentrotidae family and is mainly distributed in northern China, the Korean peninsula, the Russian far east coast, and northern Japan [17]. Although the gonads are the only edible part of sea urchins for human, *Mesocentrotus nudus* is an important mariculture species with high commercial value. Sex differences are one of the most important factors contributing to the quality of sea urchins. For instance, the testis tastes sweet and creamy, whereas the ovaries are bitter and sour [18]. The regulatory mechanisms of gonad development in sea urchins have thus attracted increasing attention in recent years [19,20]. However, sea urchins breeding has to suffer long reproductive cycle and low survival rate (range from less than 0.1 to 1%) that usually cause intractability. So far, successful gene knockout technology is unavailable to study gene function in adult sea urchin. RNA interference (RNAi), however, provides an alternative approach because of the direct injection of plasmids or RNAs. Moreover, compared to mammals, the effects of RNAi last much longer and it has been used in non-model invertebrate species, such as sea cucumber (*Apostichopus japonicus*) [21], Zhikong scallop (*Chlamys farreri*) [22], and Giant Freshwater Prawn (*Macrobrachium rosenbergii*) [23].

In the present study, we identified the *M. nudus nanos2* gene and characterized its expression patterns during embryogenesis, early ontogeny, and gonad development. Moreover, we investigated its function in gonad development by RNAi, and our data suggest that *Mnnanos2* may play an essential role in germ cell survival in adult sea urchin. This study provides insights into the functional mechanism of *nanos2* in germ cell development and sheds lights on the molecular mechanisms of gametogenesis in sea urchin.

## 2. Results

### 2.1. Isolation and Characterization of M. nudus nanos2

**Figure 1:**
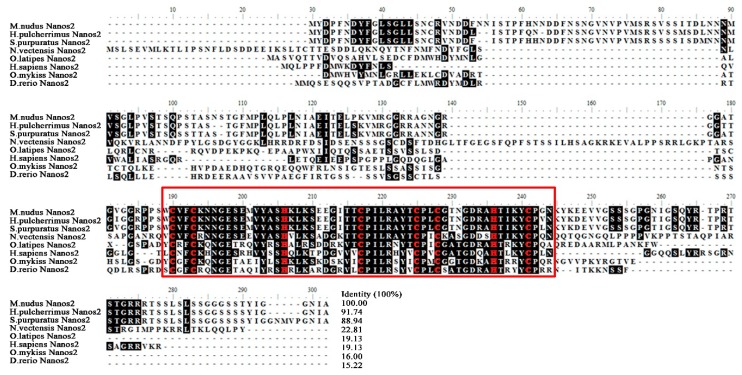
Amino acid alignments of *Mn*Nanos2 with the homologs from other species. The red box indicate the conserved posotion of zf-domain. The 8 invariant cysteine and histidine residues in zf-domin were marked with red color.

The gonad transcriptome of *M. nudus* was previously sequenced on the Illumina sequencing platform [19]. To identify the *nanos2* gene in *M. nudus*, we used the evolutionarily conserved RNA-binding zinc finger domain (ZF-domain) amino acid sequence of *S. purpuratus* NANOS2 (NM_001079555) as the query sequence, and the predicted *nanos2* gene (comp145914_c0_seq2) was identified. The *nanos2* cDNA sequence of *M. nudus* (*Mnnanos2*) was then obtained by 5′ and 3′ RACE. It is 1224-bp in length (MK577422) and contains a 693-bp open reading frame (ORF) that encodes a protein of 230 amino acids, an 84-bp 5′UTR, and a 447-bp 3′UTR. Similar to other species, *Mnnanos2* also has a highly conserved ZF-domain (aa 123–176) basing on the result of domain analysis (Figure 1).

**Figure 2:**
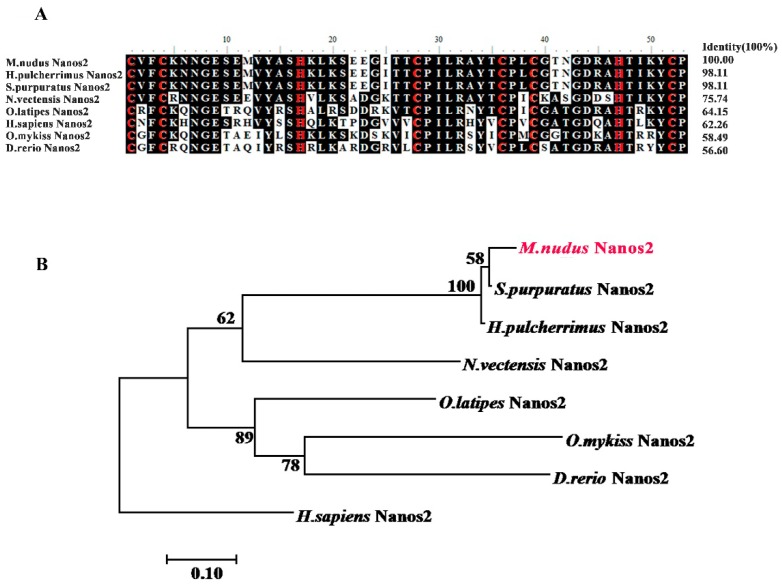
Sequence analysis of Nanos2 in *M. nudus*. (**A**) Multiple amino acid sequence alignment of Nanos2 ZF-domain, The 8 invariant cysteine and histidine residues in zf-domin were marked with red color. (**B**) phylogenetic tree of Nanos2.Nanos2 Amino acid of *Mesocentrotus nudus* was marked with red color. The phylogenetic tree was constructed with MEGA version 5.0 by bootstrap analysis using the maximum-likelihood method (1000 replicates) and human Nanos2 was used as the outgroup sequences.

Next, we downloaded eight Nanos2 protein sequences from the NCBI database and performed multiple sequence alignment analysis. As shown in Figure 1, the deduced amino acid sequence of *Mn*Nanos2 showed high identity with other echinoderm homologs (91.7% with *S. purpuratus* and 88.9% with *H. pulcherrimus*), but low identity with several other non-echinoderm homologs (only 15.2% with *D. redio*). Furthermore, the ZF-domain of *Mn*Nanos2 exhibited high levels of identity, ranging from 56.6% to 98.1%, especially with the echinoderms, including the *S. purpuratus* (98.8%) and *H. pulcherrimus* (98.8%, Figure 2A). Basing upon the multiple protein sequence alignments, a molecular phylogenetic tree was then constructed (Figure 2B). As expected, the phylogenetic tree is divided into distinct echinoderm and chordata clusters, and *Mn*Nanos2 is grouped with *S. purpuratus* and *H. pulcherrimus* Nanos2. Moreover, the topology of clades is basically consistent with the known taxonomic relationships among the species we analyzed.

### 2.2. Mnnanos2 mRNA Expression Pattern in Different Adult Tissues and Dynamic Changes during Sex Development

**Figure 3:**
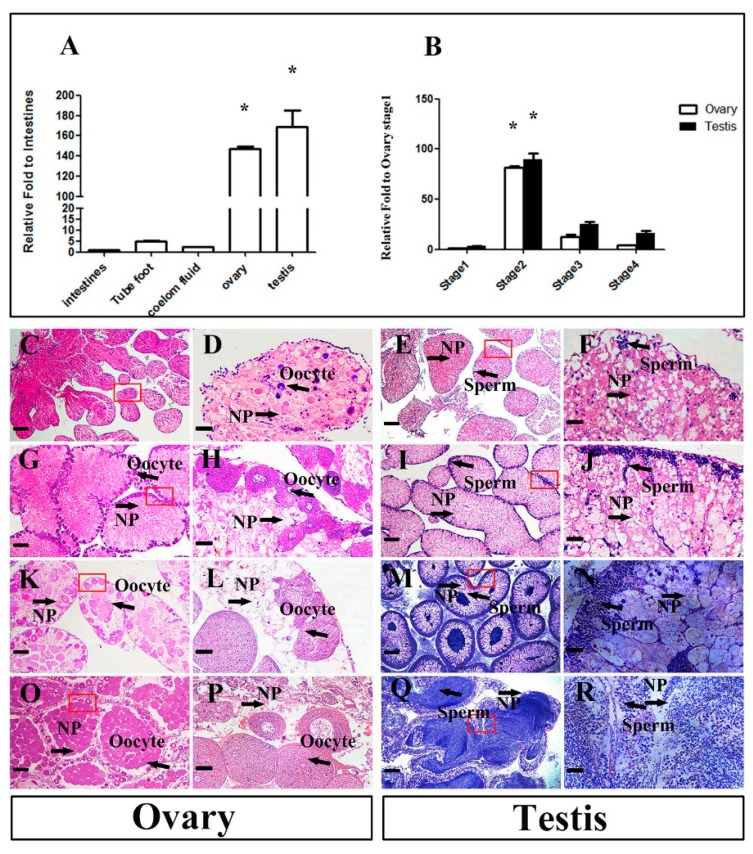
*Mnnanos2* expression in adult tissues and gonadal development stages. (**A**) RT-qPCR analysis of *nanos2* expression in different adult tissues. Ubiquitin was used as the control. The expression of *Mnnanos2* in Intestines was set to 1. Each bar represents mean ± standard deviation (SD) from three to five individuals. Tukey’s test was used to determine statistical analysis. Asterisks (*) indicate significant differences (*p* ≤ 0.05) between other tissues and intestines. (**B**) RT-qPCR analysis of *nanos2* expression at different gonadal development stages. Ubiquitin was used as the control. The expression of *Mnnanos2* at Stage 1 was set to 1. Each bar represents mean ± SD from three individuals. Data are from three independent experiments. Asterisks (*) indicate significant differences (*p* ≤ 0.05) between Stage 2–4 and Stage 1 determined by Tukey’s test. (**C**,**G**,**K**,**O**) Histological detection of the ovary at Stage 1–4. (**E**,**I**,**M**,**Q**) Histological detection of testis at Stage 1–4. The red boxed areas on the left are shown on the right at higher magnification (**D**,**H**,**L**,**P**,**F**,**J**,**N**,**R**). NP: nutritive phagocytes. Bar: (**C**,**G**,**K**,**O**,**E**,**I**,**M**,**Q**) 200 µm, and (**D**,**H**,**L**,**P**,**F**,**J**,**N**,**R**) 25 µm.

To reveal the expression pattern of *Mnnanos2*, we measured its mRNA expression by RT-qPCR in different adult tissues, including intestines, tube foot, coelom fluid, ovary, and testis. As shown in Figure 3A, *Mnnanos2* was expressed predominantly in the gonads, which is consistent with its homologs in other species. The ovary and testis of sea urchins are classified into four stages, including inter-gametogenesis and NP phagocytosis (Stage 1), pre-gametogenesis and NP renewal (Stage 2), gametogenesis and NP utilization (Stage 3), the end of gametogenesis, NP exhaustion and spawning (Stage 4) [24]. Hence, we collected the gonads at all four stages during sex development to evaluate the relative expression of *Mnnanos2*. After histological examination of the gonads (Figure 3C–R), the total RNA of ovary and testis at each stage were isolated for RT-qPCR analysis. In the ovaries, we only detected a small amount of *Mnnanos2* transcripts at inter-gametogenesis and NP phagocytosis (Figure 3B). Along with vitellogenic oocytes occurrence, the expression of *Mnnanos2* sharply increased up to 81-fold comparing to Stage 1 and reached its peak level at pre-gametogenesis and NP renewal in the ovary. Then, the *Mnnanos2* transcripts were decreased during Stage 3 and Stage 4 (6.4–25-fold lower than Stage 2) as oogenesis progressed. Similar to the ovaries, only a low level of *Mnnanos2* transcripts were found at Stage 1 of the testes. At spermatogenesis, the transcriptional levels of *Mnnanos2* dramatically increased (about 29-fold comparing to Stage 1) and reached its maximal level at Stage 2, followed by a sharp decrease at Stages 3 and 4 (3.6–5.5-fold lower than Stage 2, Figure 3B).

**Figure 4:**
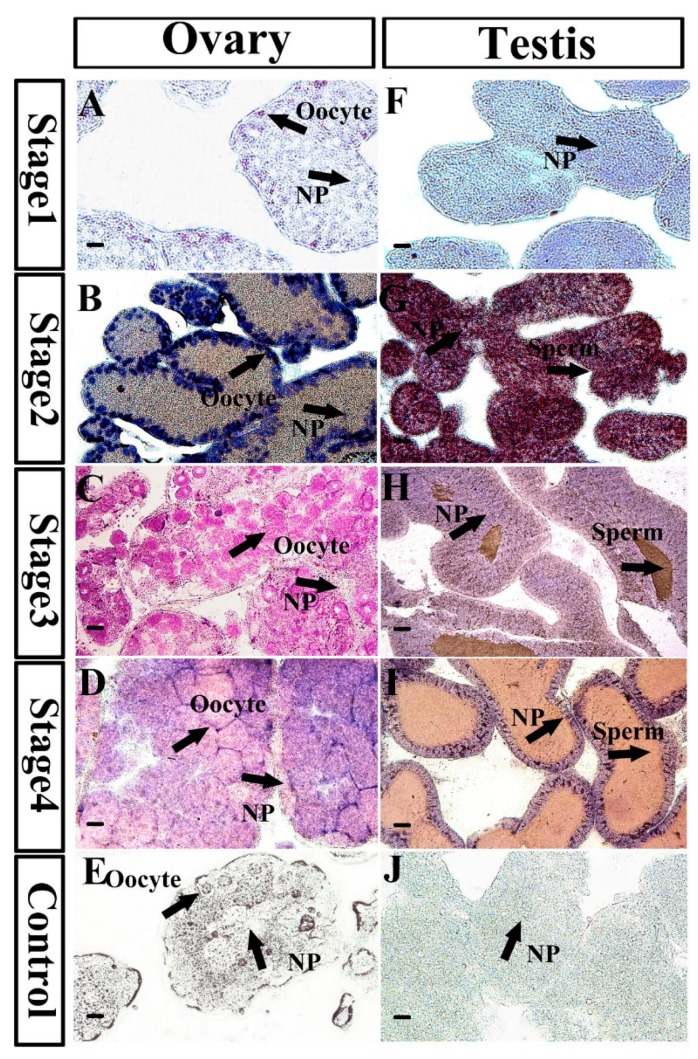
*Mnnanos2* expression analysis by in situ hybridization in gonads at different gonadal development stages. (**A**–**E**) ovary, (**F**–**J**) testis. NP: nutritive phagocytes. Bar = 100 µm.

Next, section tissue in situ hybridization analysis was performed to reveal the *Mnnanos2* cellular localization during gonad development. At Stage 1 of the ovary, *Mnnanos2* was found only in primary oocytes. During ovary development, the *Mnnanos2* transcript was continuously specifically expressed in oocytes (Figure 4A–D). At Stages 1 and 2 of the testes, *Mnnanos2* transcripts were found in all types of the gonad cells, including the germ cells and nutritive phagocytes. However, along with the testis development at Stages 3 and 4, the signals of *Mnnanos2* transcripts were most intense in NP and no positive *Mnnanos2* male germ cells were observed (Figure 4H–I). As expected, we found no positive signals in the hybridization experiments with sense probes (Figure 4E,J).

### 2.3. Mnnanos2 mRNA Expression Pattern during Embryogenesis and Early Ontogeny

**Figure 5:**
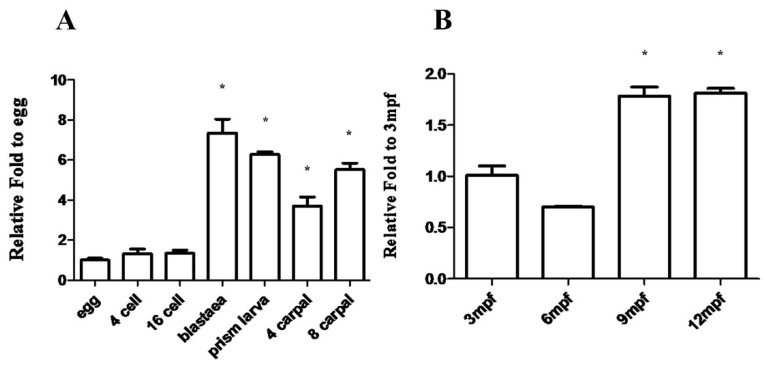
Expression analysis of *Mnnanos2* in embryogenesis and early ontogeny. (**A**) RT-qPCR analysis of *nanos2* expression in *M. nudus* embryos. Ubiquitin was used as the control. The expression of *Mnnanos2* in Egg was set to 1. Each bar represents mean ± standard deviation (SD) from more than 50 individuals. Asterisks (*) indicate significant differences (*p* ≤ 0.05) between 4 cell—8 carpal and egg (**B**) RT-qPCR analysis of *nanos2* expression in early ontogeny of *M. nudus*. Ubiquitin was used as the control. The expression of *Mnnanos2* in 3 mpf was set to 1. Each bar represents mean ± SD from three individuals. Data are from three independent experiments. Asterisks (*) indicate significant differences (*p* ≤ 0.05) between 6–12 mpf and 3 mpf. Mpf: months post-fertilization. Tukey’s test was used to determine statistical analysis.

Since abundant *Mnnanos2* transcripts were detected in the oocytes, we further detected the expression patterns of *Mnnanos2* during embryogenesis and early ontogeny by RT-qPCR. Consistent with previous results in *S. purpuratus* [16], *Mnnanos2* transcripts were detected at early stages from the egg to 16 cells (Figure 5A). At blastula, the expression level of *MnNanos2* was significant increased and continuously expressed during prism larva, four carpal and eight carpals (Figure 5A). With ontogenesis, the transcripts of *Mnnanos2* could still be detected at three months post-fertilization (mpf), when the shell diameter of *M. nudus* is about 8 ± 2 mm (Figure 5B). The expression level of *Mnnanos2* decreased subsequently at 6 mpf (shell diameter: 10 ± 2 mm, 1.45-fold comparing to 3 mpf), but then sharply increased at 9 and 12 mpf (shell diameters: 13 ± 2 mm and 14 ± 2 mm, respectivley, >1.9-fold comparing to 3 mpf).

### 2.4. Characterization of MnNanos2 Protein Expression Pattern in Gonads

**Figure 6:**
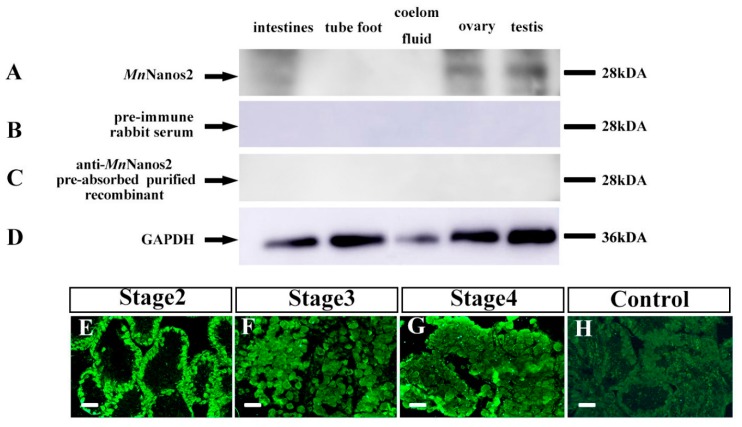
Detection of anti-*Mn*Nanos2 antibody and immunofluorescence localization of *Mn*Naons2 in the ovary. (**A**) Western blot with the anti-*Mn*Nanos2 polyclonal antibody in ovary and testis extracts, (**B**) Western blot with the pre-immune rabbit serum in ovary and testis extracts, (**C**) Western blot with the anti-*Mn*Nanos2 antibody that had been pre-absorbed with the purified recombinant *Mn*Nanos2 protein in ovary and testis extracts, (**D**) GAPDH was used as the control, (**E**–**G**) immunofluorescence localization of *Mn*Nanos2 in the ovary, (**H**) immunofluorescence localization of pre-immuned serum in the ovary. Bar = 100 µm.

To further describe the protein expression and cellular localization of *Mn*Nanos2 in gonads, we prepared the anti-*Mn*Nanos2 polyclonal antibody in vitro and performed Western blot detection. We examined the antibody specificity by comparing the pre-immune serum with a pre-adsorbed anti-*Mn*Nanos2 polyclonal antibody. Then, we measured its protein expression by western blotting in different adult tissues, including intestines, tube foot, coelom fluid, ovary, and testis. As shown in Figure 6A, the anti-*Mn*Nanos2 polyclonal antibody specifically recognized a polypeptide at around 28 kDa (the predicted molecular weight of MnNanos2 is 25.41 kDa) in both the ovary and testis extracts. Blotting with pre-immuned serum (serum incubated with *Mn*Nanos2 protein prior to immunoblotting) led to a complete disappearance of this 28 kDa band (Figure 6B). Likewise, when the anti-*Mn*Nanos2 polyclonal antibody was pre-adsorbed with the purified recombinant *Mn*Nanos2 protein at 4 °C for 16 h, the specific 28-kDa polypeptide could not be detected by the pre-adsorbed antibody (Figure 6C). Meanwhile, GAPDH was used as the control (Figure 6D) and the full uncut labeled blots can be found in Figure S1. These data indicate that the anti-*Mn*Nanos2 polyclonal antibody is specifically expressed in gonads. Furthermore, immunohistochemistry analysis was performed to confirm the cellular localization of *Mn*Nanos2 proteins. Consistent with the ISH results, *Mn*Nanos2 protein was detected in oocytes throughout the development of the ovary (Figure 6E–G). As expected, we found no positive signals in the ovary that were incubated with pre-immuned serum (Figure 6H). However, we could not detect the positive signals in testis, Stage 1 ovary (data not shown) and intestines (Figure S2). According to previous studies, many factors including tissue, duration, and type of antigen retrieval can affect the result [25].

**Figure 7:**
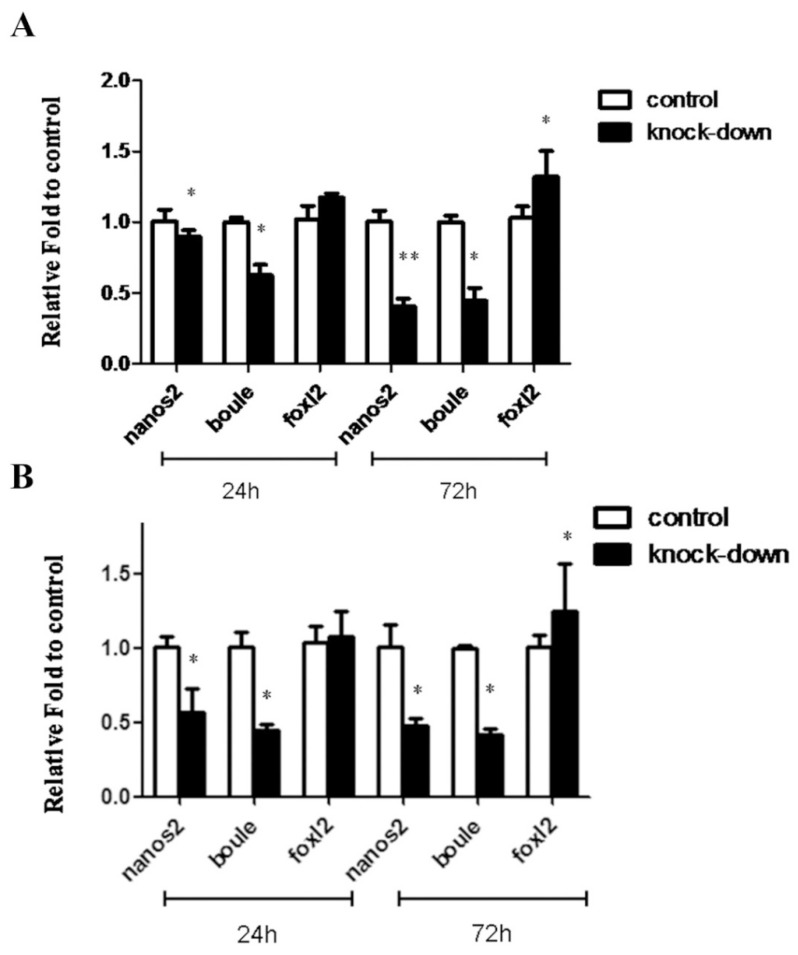
RNA interference (RNAi) of *Mnnanos2*. RT-qPCR measured the mRNA levels of *Mnnanos2* and other sex-related genes after *Mnnanos2* RNAi. (**A**) ovary and (**B**) testis. Ubiquitin was used as the control. The expression of *Mnnanos2* in control group was set to 1. Each bar represents mean ± standard deviation (SD) from three individuals. Asterisks (*) indicate significant differences (*p* ≤ 0.05) between knockdown and control. The datasets were normal distribution by Shapiro–Wilk test. Student’s test was used for statistical analysis.

Considering that *Nanos2* plays a conserved role in germ cell development [6] and that the *MnNanos2* gene is expressed predominantly in gonads, we utilized RNAi to investigate *MnNanos2* function in gonad development. Adult *M. nudus* were injected with either specific *MnNanos2*-targeting double-stranded RNA (dsRNA) or phosphate buffer saline (PBS), and were then divided into the knockdown and control subgroups, respectively. Then we collected gonadal samples at 24 and 72 h after injection to measure the expression of *Mnnanos2* and other well-studied sex-related genes, including *boule* [26] and *foxl2* [27], by RT-qPCR. As shown in Figure 7A, the expression of *boule* in the ovary significantly decreased in *Mnnanos2*-knockdown samples comparing to the control groups at 24 h after injection. However, the transcripts of *Mnnanos2* and *foxl2* showed quantitatively small differences at the same time point. At 72 h post injection, the expression of *Mnnanos2* and *boule* significantly decreased by 59.6% and 56.0% in the *Mnnanos2-*knockdown group comparing to the control group. Interestingly, the *foxl2* expression level increased about 1.3-fold in the ovary of the knockdown group at 72 h post injection. In the testes, *Mnnanos2* and *boule* expression were decreased by 44.0% and 55.9% at 24 h after injection in knockdown adults, respectively. The transcripts of *Mnnanos2* and *boule* were similarly suppressed significantly (by 53.4% and 58.7%, respectively) in male knockdown adults at 72 h after injection. In addition, the *foxl2* expression in male *MnNanos2*-knockdown adults phenocopied that of the ovaries at 72 h after injection (Figure 7B).

## 3. Discussion

As the only edible part of *M. nudus*, the gonads have dual functions of reproduction and nutrient storage [17]. Therefore, understanding the regulatory mechanisms of gonad development is of great theoretical and practical significance. In this study, we identified the *nanos2* gene homolog in *M. nudus* and revealed its molecular characterization, phylogenetic relationships, and sexually dimorphic expression. Importantly, we studied the *nanos2* gene function in adult sea urchins with RNAi.

We detected *Mnnanos2* in different stages of gonads. Interestingly, the expression level of *Mnnanos2* reached its peak at Stage 2 in both the ovary and testis (Figure 3B). This highest mRNA expression at Stage 2 of ovary and testis might be involved in gametogenesis because a large number of oocytes and spermatogenic cells must be produced at the pre-gametogenesis and NP renewal stage. Furthermore, *Mnnanos2* was expressed continuously and specifically in oocytes of the ovary at different stages, and *Mnnanos2* could thus be a marker of oocytes in sea urchins (Figure 4A–D). The specific expression of *Mnnanos2* in oocytes indicates that it plays an essential role in regulating oocyte production. However, this expression pattern is different from previous reports about *nanos2* in vertebrates. For example, *nanos2* is specifically detected in germ stem cells of the ovary in zebrafish [9], medaka [28], and orange spotted grouper [5]. Interestingly, *Mnnanos2* was expressed in both the male germ cell and NP cells at Stage 2 of the testis (Figure 4G) and was continuously presented in NP cells during the gonad Stages 3 and 4 (Figure 4H–I). However, this expression pattern is remarkably different from other species. For instance, in inchoate buffalo and trout, the highest *nanos2* mRNA expression is detected in testes and is mainly expressed in undifferentiated type A spermatogonia, thus it should be a molecular marker of spermatogonia [4,13,29]. Moreover, *Mnnanos2* was found in embryos, which is similar to *Caenorhabditis elegans* [30,31], *Strongylocentrotus purpuratus* [15], and mouse [8,32], but different from most teleost species, in which no *nanos2* mRNA was observed during embryogenesis [9,19,28]. This differential pattern of expression may indicate that the gene has gone through functional divergence during millions of years of evolution.

Recently, knock-off [33] and knockdown [34] technologies have been used in sea urchins, but all of these studies have focused on embryonic development. Here, we tried to uncover the function of *nanos2* gene in adult gonads by means of RNAi. By injecting sea urchin with *nanos2* dsRNA, we successfully reduced the expression of not just our target gene, but also other conserved germ cell marker genes like *boule* (Figure 7). In vertebrates as well as invertebrates, *nanos2* and *boule* have been identified as germ cell markers and play important roles in germ cell development [12,35]. The decreased expression of germ cell marker genes (*nanos2* and *boule*) indicated that the number of germ cells may have decreased, and that *Mnnanos2* is essential for germ cell survival in adult sea urchin. Interestingly, the expression level of *foxl2* was increased slightly, rather than decreased, in the knockdown ovary and testis comparing to that of the control group. In most species, *foxl2* is predominantly expressed in ovarian granulosa cells and is required for ovary differentiation and maintenance [36,37,38,39,40]. Recently, it has been reported that the *foxl2* transcript is expressed in both the ovary and testis of *M. nudus* [19]. However, the mechanism of this increased expression of *foxl2* in *nanos2*-knockdown sea urchins is unclear and should be further studied. These above data suggest that dsRNA injection is an effective method to study gene function in adult sea urchins and that more functional studies need to be carried out on these genes to determine their molecular mechanisms in sea urchin sex development.

In conclusion, we identified the *nanos2* gene homolog in *M. nudus* and show that *Mnnanos2* is a maternal factor that can be detected continuously during embryogenesis and early ontogeny. *Mnnanos2* displays sexually dimorphic characteristic in gametogenesis, and it might be a germ-cell-specific marker in the ovary. In testis, *Mnnanos2* is expressed both in NP cells and male germ cells, which is different from other species. In addition, the expression level of *nanos2* and *boule* were significantly decreased while *foxl2* was increased in *nanos2* knockdown sea urchins by RNAi. In the future, the molecular mechanism of *nanos2* in regulating germ cell development should be further investigated in sea urchins.

## 4. Materials and Methods

### 4.1. Experimental Animals

*M. nudus* used in the experiment were collected from the coastal areas of Dalian, China (38.8073 N, 121.4045 E). The samples of early embryogenesis were obtained from Key Laboratory of Mariculture & Stock Enhancement in North China Sea, Ministry of Agriculture and Rural Affairs. This study did not involve any endangered or protected species.

### 4.2. Gonadal Histology

Gonadal tissues were surgically removed and fixed in 4% paraformaldehyde (PFA) at 4 °C overnight and embedded in Optimal Cutting Temperature (O.C.T). at room temperature. Then, sections (3 µm) were cut and stained with hematoxylin/eosin (HE; Beyotime Institute of Biology, Suzhou, China). The gonadal phase was observed in Zeiss microscopy.

### 4.3. RNA Extraction and Real-Time Quantitative PCR (RT-qPCR)

Total RNAs was extracted from various tissues, including the intestines, coelom fluid, tube foot, ovary, and testis by using SV Total RNA Isolation System (Promaga Z3100). Embryos during different development stages, including egg, 4 cell, 16 cell, blastula, prism larva, 4 carpal and 8 carpals were collected to extract total RNAs (*n* > 50). Moreover, the gonads of early ontogeny were surgically removed under Zeiss microscopy to extract total RNAs. Total RNAs (1 µg) of ovary and testis were used to establish cDNA library by using the SMARTer™ RACE cDNA Amplification Kit (Clontech 634923) according to the manufacture’s protocols. Besides, 1 µg of total RNAs were used to synthesize the first-strand cDNAs based on the protocols of GoScriptTMReverse Transcription System (Promega A5000).

RT-qPCR experiments were performed in 20 µL reactions containing 2 µL of cDNA that had been diluted 5 times, 0.8 µL of each 10 mM primer, 10 µL of Fast Start Essential DNA Green Master (Roche, Mannheim, Germany) and 6.4 µL sterile water. The protocol was 95 °C (10 min) for heat denaturing, then, 40 cycles of 95 °C (15 s), 60 °C (1 min). All primers used were designed by the software of Primer5 (Table S1). The efficiency of primers discovery through standard curve formulation reached 96%. Ubiquitin was used as an internal control [41]. The target genes relative expression was calculated with the 2^—ΔΔ*C*t^ method. For statistical analysis, Tukey’s test and Student’s *t*-test were calculated with SPSS software after normal distribution and homogeneity of variance test (SPSS Inc.). A probability (P) of <0.05 was considered statistically significant.

### 4.4. Sequence and Phylogenetic Analyses

The full-length Mn*nanos2* cDNA was achieved by 5′ and 3′ rapid amplification of cDNA ends (RACE). The DNAMAN software was used to predict the deduced amino acid sequences. Multiple protein sequence alignment was performed by Clustal W. The phylogenetic tree was constructed with MEGA version 5.0 by bootstrap analysis using the maximum-likelihood method (1000 replicates) and human NANOS2 was used as the outgroup sequences.

### 4.5. In Situ Hybridization

Gonad samples were fixed in 4% PFA at 4 °C overnight. Then, embedded in OCT and sections (10 µm) were cut with frozen microtomy. Primers used for amplifying antisense and sense RNA probe templates containing a T7 RNA polymerase promoter were designed by the software of Primer5 (Table S1). After PCR amplification and PCR product recovery, the recovered purified PCR products were used as the templates for probes. Subsequently, the riboprobes were synthesized by using digoxigenin (DIG) RNA labeling kit (Roche) according to the manufacturer’s protocol. Section tissues in-situ hybridization was carried out according to the previously described [42]. Digital images were photographed using a (Lecia DM4B) microscope.

### 4.6. dsRNA Synthesis and Injection

The gene-specific dsRNA was designed by https://www.dkfz.de/signaling/e-rnai3/ and synthesized with MEGAscript RNAi Kit (Thermo Fisher, Vilnius, Lithuania) according to the protocol. A total of 24 *M. nudus* samples with a mean shell diameter of 48.3 ± 2 mm were randomly assigned to the control group and the knockdown group. Subsequently, 50 µL PBS and 50 µL PBS containing 0.631 µg *nanos2* dsRNA were injected into sea urchins of the control group and the knockdown group, respectively. On the 24 h and 72 h after injection, three male sea urchins and three female sea urchins from each group were removed randomly, and their gonads were sampled to analyze dynamic expression changes of the germ cell marker genes.

### 4.7. Western Blot Analysis

The gonads at the pre-gametogenesis and NP renewal stages were collected and digestion with 300 µL radio-immunoprecipitation assay lysis buffer (RIPA) containing 1% protease inhibitor cocktail (Roche). Extracts were then resolved by 4–12% Sure PAGE (GenScript, Piscataway, NJ, USA), electroblotted onto Immobilon ^®^-P transfer membranes. Western blotting used primary anti-Nanos2 rabbit antibodies (1:100). This was followed by incubation with goat anti-rabbit IgG (1:2000). The detection of immunoreactivity was by using the Immobilon TM Western HRP Substrate Luminol Reagent.

### 4.8. Immunofluorescence Localization

Gonads were fixed overnight in 4% paraformaldehyde, rinsed, and balanced in 30% sugar. Samples were embedded in OCT and frozen in liquid nitrogen and stored at −80 °C until use. Serial sections were 7-µm thick on poly-l-lysine-coated glass slides. The tissue was rehydrated with PBS, then sealed with 5% non-fat powdered milk and 0.5% Triton for 1 h. Subsequently, gonads were incubated overnight at 4 °C with the primary anti-Nanos2 antibody (1:50) followed by incubation with goat anti-rabbit IgG Alexa Fluor TM Plus 488 (1:200). Digital images were taken on (Lecia DM4B) microscopes.

## Supplementary Materials

Supplementary materials can be found at https://www.mdpi.com/1422-0067/20/11/2705/s1.
