# Supplementary material for: Molecular Cloning and Sexually Dimorphic Expression Analysis of nanos2 in the Sea Urchin, Mesocentrotus nudus
Source: Int J Mol Sci. 2019 Jun 1;20(11):2705. doi: 10.3390/ijms20112705 (PMC6600436; doi:10.3390/ijms20112705)
Supplement: Supplementary file 1 [file ijms-20-02705-s001.pdf]

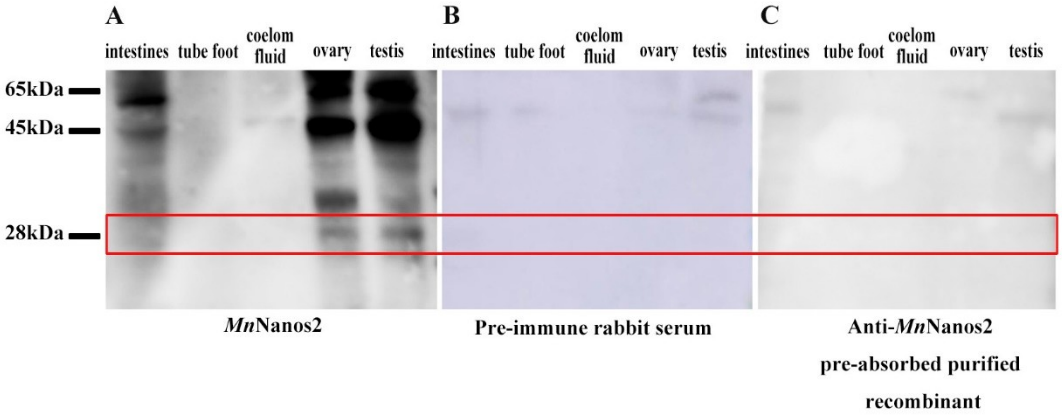

Figure S1. (A) Western blot with the anti-*Mn*Nanos2 polyclonal antibody in ovary and testis extracts; (B) Western blot with the pre-immune rabbit serum in ovary and testis extracts; (C) Western blot with the anti-*Mn*Nanos2 antibody that had been pre-absorbed with the purified recombinant *Mn*Nanos2 protein in ovary and testis extracts.

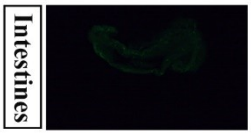

Figure S2. immunofluorescence localization of *Mn*Nanos2 in the intestines.

Table S1. Sequences of the primers used for experiment.

Primer name	Purpose	Sequence(5′ to 3′)
Nanos2-F1	ORF	TTGTCACCATGTACGACC
Nanos2-R1	ORF	GTGCTGCAAAGGCTGCTC
Nanos2-F2	5′RACE-1	AACGGTGTCGAGATAT
Nanos2-F3	5′RACE-2	AAAGTCATCGTTCACTCTGC
Nanos2-F4	5′RACE-3	CAACAGTCCACTGAGTCCA
Nanos2-R2	3′RACE-1	CGGATCCCAGTATAGAACCCCACG
Nanos2-R3	3′RACE-2	CTCTGGCGGAGGTTCATCTACTTA
Nanos2-F5	RT-PCR	CTATGCGGAACGAACGGCGACC
Nanos2-R4	RT-PCR	GTCGGTGTCGTTGGCTAGCGAAG
Ubiquitin -F	RT-PCR	TGGTCGCACTCTCTCAGACTACAAC
Ubiquitin -R	RT-PCR	TGCCGTCTCTCAATTTGTCTATAGC
Nanos2 –F6	Situ hybridization	ACACCGTTCCACAACAATG
Nanos2 –R6	Situ hybridization	TAATACGACTCACTATAGGTCAAGTAGATGAACCT CCGCCAG
ds –RNA F	RNAi	TAATACGACTCACTATAGGGTATCTCGACACCGTT CCACA
ds –RNA R	RNAi	TAATACGACTCACTATAGGGAAGTAGATGAACCTCC GCCA
